# Age of mother and grandmother in relation to a subject's breast cancer risk

**DOI:** 10.1038/sj.bjc.6605639

**Published:** 2010-03-30

**Authors:** M C de Haan, K B Michels, P H M Peeters, P A H van Noord, F A M Hennekam, Y T van der Schouw

**Affiliations:** 1 Julius Center for Health Sciences and Primary Care, University Medical Center Utrecht, Room STR 6.131, PO Box 85500, 3508 GA Utrecht, The Netherlands; 2 Department of Obstetrics, Gynecology and Reproductive Biology, Obstetrics and Gynecology Epidemiology Center, Brigham and Women's Hospital, Harvard Medical School, 221 Longwood Avenue, Boston, MA 02215, USA; 3 Department of Epidemiology, Harvard School of Public Health, Boston, MA 02115, USA; 4 Department of Medical Genetics, University Medical Center Utrecht, Utrecht, The Netherlands

**Keywords:** maternal age, grandmaternal age, breast cancer risk, oocyte

## Abstract

**Background:**

On theoretical grounds, the age of the grandmother and the age of the mother at delivery of her daughter may affect the breast cancer risk of the granddaughter.

**Methods:**

We used the data relating to the Diagnostic Research Mamma-carcinoma cohort (DOM (Diagnostisch Onderzoek Mammacarcinoom) 3), which comprises a population-based sample of 12 178 women aged 41–63 years at enrolment in 1982–85 and followed up until 2000. During follow-up 340 postmenopausal breast cancer cases were identified. To these we applied a case–cohort design together with a random sample from the baseline cohort (*n*=1826). Of these study participants, we were able to retrieve the birth dates of 998 mothers (309 cases, 689 controls), and for 547 of these we also retrieved the birth dates of the grandmothers (197 cases, 350 controls). A weighted Cox proportional hazards model was used to estimate the hazard ratios (HRs) for the effect of the age of the grandmother and the age of the mother on the breast cancer risk of the index women, while adjusting for potential confounders.

**Results:**

Compared with the reference group aged 25–29.9 years, the group with the lowest maternal age (<25 years) had an age-adjusted HR of 0.77 (95% CI 0.19–3.12) and the group with the highest maternal age (⩾40 years) had an age-adjusted HR of 1.58 (95% CI 0.01–267.81), *P*-value for trend=0.62. Compared with the same reference group, the group with the lowest grandmaternal age (<25 years) had an age-adjusted HR of 0.53 (95% CI 0.24–1.17) and the group with the highest grandmaternal age (⩾40 years) had an age-adjusted HR of 7.29 (95% CI 1.20–44.46), *P* for trend=0.04. The associations did not change significantly after additional adjustment for various risk factors for breast cancer, neither for maternal age nor for grandmaternal age.

**Conclusion:**

This study does not suggest a major role of maternal age at delivery or grandmaternal age at delivery of the mother for the (grand)daughters' breast cancer risk.

Breast cancer is the commonest cancer in women worldwide. Several studies show breast cancer risk to increase with characteristics that can be considered as proxy of early-life events, such as higher paternal and maternal age (Xue *et al*, 2007).

Besides hypotheses on the role of post-conception (intrauterine) early-life events, a pre-conception hypothesis on the quality of the oocyte during conception as a risk factor for adult breast cancer has also been put forward (Trichopoulos, 1990; van Noord, 2002).

The viability of the oocyte seems to be co-determined by the quality of its mitochondria and their energy/ATP production, which declines with age (Papa, 1996; Ozawa, 1997).

Suboptimal energy production by less viable mitochondria may, for example, affect the correct functioning of the cell spindle required for proper chromosome separation during the first five cell divisions. Aberrant spindle function may result either in spontaneous abortion, or, when the fertilised egg survives, in (a higher propensity to) aneuploidy in the fetus and related birth defects which might not be directly noticeable at birth.

Mitochondria are passed on from mother to child only; therefore, a high maternal age (resulting in less viable mitochondria) might increase the risk of several cancers in the offspring (Deusberg *et al*, 1998; Van Noord, 2002, 2003). Consequently, there may be trans-generational effects, because mitochondria are passed on from mother to daughter to granddaughter (Van Noord-Zaadstra *et al*, 1991).

We explored whether there is a relation between grandmother's age at birth of the mother and breast cancer risk of the index woman, and whether there is a relation between the mother's age at birth of the index woman, and breast cancer risk of the index woman, using data from the Dutch DOM (Diagnostisch Onderzoek Mammacarcinoom) cohort.

## MATERIALS AND METHODS

The DOM cohort consists of 55 519 women, born between 1911 and 1945, who participated voluntarily in a population-based breast cancer screening project (DOM project). The participants were recruited in Utrecht and the surrounding municipalities in the Netherlands between 1974 and 1986. Depending on their year of birth they were included in one of the four distinct cohorts, each of which completed different questionnaires. For the purpose of this study, we used the data of the third cohort consisting of 12 178 women, born in 1932–41 and recruited in 1982–85. For these women data were available on early-life characteristics and on the most important reproductive risk factors for breast cancer. The design and methodology of the DOM project has been described previously (De Waard *et al*, 1984). To prevent a costly and time-consuming assessment of vital status during follow-up, the efficiency of a case–cohort study design was chosen, in which a random sample of the total cohort is used to represent the total person-years lived for the entire cohort (Barlow *et al*, 1999). We selected at random a sample of approximately 15% of the total cohort (*n*=1826), which was followed up until 1 January 2000, by using regional municipality registries for mortality and movement out of the catchment area of the cancer registry.

In the Netherlands, the Central Bureau for Genealogy (CBG) preserves the personal data of deceased persons. These data were primarily collected by the municipal registries: this was by hand on a hardcopy card from 1938–39 onwards until this was computerised in 1994. For each Dutch citizen born before 1994 a personal hardcopy card is stored containing the name, date of birth, nationality, names and birth dates of the parents. In 1994 the personal hardcopy card was replaced by a digital personal record. All persons still living have a digital personal record, which is not yet available at the CBG. We collected these data of the DOM participants using the electronic database of the national municipal administration registration (GBA). In addition, we collected the personal record cards of the mother of the DOM participants, containing the birth dates of the grandparents.

Maternal and grandmother's age at delivery were calculated by subtracting the DOM woman's date of birth from the (grand)maternal date of birth. Women with missing data on year of birth or on the year of birth of their (grand)mother were excluded. Through genealogical research we were able to retrieve the birth dates of 998 mothers (309 cases, 689 controls) and 547 grandmothers (197 cases, 350 controls). For 547 subjects (197 cases, 350 controls), we had complete maternal and grandmaternal birth data. The study was approved by the Institutional Review Board of UMC Utrecht.

At recruitment, 72% of the participants were not yet postmenopausal. During follow-up, for some women updated information on menopausal status and age was collected, and this information was used in the analysis. If it was missing (*n*=436) we imputed the menopausal status at age 52 for smokers and at age 55 for non-smokers (Parente *et al*, 2008). Pre-menopausal breast cancer cases were excluded from the analyses.

We identified all primary invasive breast cancers (*n*=435) that occurred until 1 January 2000 in the DOM-3 cohort. The DOM project started as a mammographical breast cancer screening project, which was set up to assess its effect on breast cancer occurrence and mortality. For this an active registration team was set up, evolving into the regional cancer registry, which has become complete for the detection of all cancers from 1987 onwards. It is one of the eight comprehensive cancer centres in the Netherlands that together form the national cancer registry that is part of the international databank of the IARC (International Agency of Cancer Research) and the European databank of the ENCR (European Network Cancer Registration) (http://www.ikc.nl). From 1989 onwards, the DOM database is linked to the regional cancer registry on an annual basis to identify all new breast cancer cases within the DOM cohort.

Many potential risk factors for breast cancer were assessed through the questionnaires. As in other studies, the risk factors we considered were as follows: age, premature birth, birth order (firstborn), birth weight and birth length, adult height, weight at age 18, current weight at baseline, alcohol consumption, physical activity in leisure time, age at menarche, age at menopause, menopausal status at diagnosis, ever oral contraceptive use, ever postmenopausal hormone use, age at first birth, parity, history of benign breast disease and family history of breast cancer among first-degree relatives. In addition, we also considered the number of months of full lactation during the lifetime (Xue and Michels, 2007).

### Statistical analysis

To establish the possible association between breast cancer risk of the (grand)daughter and (grand)maternal age at the time her daughter was born, we used weighted Cox regression analyses. The methods for these analyses are largely similar to a standard Cox regression and are well described (Barlow *et al*, 1999). Follow-up time ended at the date of the breast cancer diagnosis. Women who remained free of breast cancer during the observation period were censored at the date of movement, date of death or on 1 January 2000, whichever occurred first. Analyses were done with SAS version 8.2 by use of a weighted Cox regression macro (available at http://lib.stat.cmu.edu/general/robphreg) that computes the weighted hazard ratios together with robust s.e.'s, which we used to calculate the 95% confidence intervals. We fitted both age-adjusted and covariate-adjusted Cox proportional hazard models.

We analysed the effect of maternal age on breast cancer in 998 DOM participants by fitting four models: in the first we adjusted for age only, the second takes account of age and all breast cancer risk factors, the third adjusts for age and age of the grandmother (*n*=547 women), the final model adjusts for age, age of the grandmother and all breast cancer risk factors (*n*=547). We analysed the effect of grandmaternal age on breast cancer risk in the granddaughter by fitting again four models as above, now in 547 DOM women. Both maternal and grandmaternal ages were categorised as <25, 25–29.9, 30–34.9, 35–39.9 and ⩾40 years. We chose grandmaternal age 25–29.9 years as the reference group in all models, because this was the largest group in both variables. Trend tests were performed with midpoints of categories.

The covariate-adjusted model included the characteristics of intrauterine exposures, namely birth weight, birth length, prematurity and firstborn. Furthermore, this model was adjusted for the following risk factors: alcohol (categorised as never, sometimes or often), BMI (categorised as <20, 20–25, 25–30, 30–35 and ⩾35 kg m^−2^), postmenopausal HRT use (ever/never), a positive history of benign breast disease (dysplasia) (yes/no), a positive family history of breast cancer (yes/no) and age at first delivery (categorised as <25, 25–29.9 and ⩾30 years or as an indicator variable if nulliparous), parity (categorised as 0, 1, 2, 3 or ⩾4 years) and age at menopause (continuous). Missing data for each covariate were analysed as indicator variables in the model.

## RESULTS

In 1982–2000, 435 breast cancer cases occurred in the whole DOM3 cohort (*n*=12 178), of which 340 cases were postmenopausal. The 1826 participants in the random sample, including all breast cancer cases, contributed 25 906 person-years. The distribution of most personal characteristics of the participants for whom we knew the age of the mother (*n*=998) did not differ across the categories of maternal age at delivery, but women whose mother was older at delivery were more likely to be prematurely delivered, to have a family history of breast cancer, to be slightly older at first childbirth, to have a slightly lower BMI, and to drink more alcohol, and less likely to be firstborn (Table 1). The distribution of these characteristics among the participants for whom we knew the age of the grandmother (*n*=547) showed the same pattern, except for family history, which was less likely to be present when the grandmother was older at delivery.

The mean maternal age at delivery was 30.5 years, with a minimum age of 16.8 and a maximum age of 47.9 years. The mean grandmaternal age at delivery of the mother of the index woman was 31.0 years, with a minimum age of 18.4 years and a maximum age of 50.8.

In age-adjusted analysis, maternal age was not associated with breast cancer risk (*P* for trend=0.62). When compared with the reference group of maternal age 25–29.9 years, the group with the lowest maternal age (<25 years) had an age-adjusted hazard ratio (HR) of 0.77 (95% CI 0.19–3.12), and the group with the highest maternal age (⩾40 years) showed a HR of 1.58 (95% CI 0.01–267.81) (Table 2). The association did not change after additional adjustment for characteristics as proxy for intrauterine exposures or breast cancer risk factors (*P* for trend=0.76).

Grandmaternal age showed a borderline significant association with the breast cancer risk of the granddaughter after adjustment for age (*P* for trend=0.05). When compared with the reference group of grandmaternal age 25–29.9 years, the group with the lowest grandmaternal age (<25 years) had an age-adjusted HR of 0.53 (95% CI 0.24–1.17) and the group with the highest grandmaternal age (⩾40 years) had an age-adjusted HR of 7.29 (95% CI 1.20–44.46) (Table 2). After adjusting for further confounders, the association between grandmaternal age and breast cancer risk was no longer significant (*P* for trend=0.89); HR for the group with the highest maternal age (⩾40 years) was 1.22 (95% CI 0.25–5.92).

When maternal and grandmaternal age were combined in one model, there was again no evidence for a relation between grandmother's age at birth of the mother and breast cancer risk of the index woman, nor for a relation between the mother's age at birth of the index woman and breast cancer risk of the index woman. Owing to the limited sample size, we were not able to stratify for the birth order, menopausal status, or family history.

We did not have detailed nutritional information of the participants, but we did ask for daily milk consumption and for the type of milk that was consumed (categorised as <1 per day, 1–2 per day or >2 per day and categorised as no milk, skimmed milk, half-full milk, varies, or full-fat milk). Although relations with breast cancer are not consistent (Cho *et al*, 2003), we also ran models including this variable, but the effects did not change.

## DISCUSSION

In this prospective cohort study, we did not observe an association between grandmaternal or maternal age and breast cancer risk among their granddaughters. To our knowledge, this is the first study, in which the association between grandmothers' age at delivery of the mother and breast cancer in the daughter is explored. At least 7 cohort studies (Holmberg *et al*, 1995; Zhang *et al*, 1995; Mogren *et al*, 1999; Hemminki and Kyyronen, 1999; Hilakivi-Clarke *et al*, 2001; McCormack *et al*, 2003; Xue and Michels, 2007) and 14 case–control studies (see below for references) have examined maternal age at delivery in relation to breast cancer risk, with inconsistent results. The largest cohort study found a positive relation for women with mothers’ age over 40 but no linear trend between the age of the mother and breast cancer (Holmberg *et al*, 1995). The second largest study observed a moderate positive relation with maternal age (Xue and Michels, 2007), whereas two studies found a non-significant positive association (Zhang *et al*, 1995; Mogren *et al*, 1999). The other three studies showed no association with maternal age (Hemminki *et al*, 1999; Hilakivi-Clarke *et al*, 2001; McCormack *et al*, 2003). Of the 14 case–control studies, 5 report a significant positive association between maternal age and breast cancer (Rothman *et al*, 1980; Janerich *et al*, 1989; Thompson and Janerich, 1990; Innes *et al*, 2002; Hodgsen *et al*, 2004), 3 studies report a non-significant positive association (Ekbom *et al*, 1997; Choi *et al*, 2005; Park *et al*, 2006) and 6 studies do not find an association between maternal age and breast cancer risk (Le Marchand *et al*, 1988; Sanderson *et al*, 1996; Newcomb *et al*, 1997; Weiss *et al*, 1997; Titus-Ernstoff *et al*, 2002; Mellemkjaer *et al*, 2003). Reasons for the inconsistencies in results may include the variety in study designs or study populations.

Several of these studies assessed maternal age by recall of the daughter, which is likely to be less accurate than through birth certificates as in our study. In addition, some studies only had information on characteristics that we consider a proxy for intra-uterine exposure, but lacked information on risk factors acting later in life. Two cohort studies collected prospective information about adult risk factors for breast cancer through questionnaires, in addition to retrospective information (Zhang *et al*, 1995; Xue and Michels, 2007); both found a positive relation with maternal age. The remaining cohort studies mainly used retrospective information regarding childhood factors in their analyses and were therefore not able to adjust for adult risk factors (Holmberg *et al*, 1995; Hemminki and Kyyronen, 1999; Mogren *et al*, 1999; Hilakivi-Clarke *et al*, 2001; McCormack *et al*, 2003).

As we were unable to find any association between (grand)maternal age and breast cancer risk in the offspring, our hypothesis may be ‘incorrect’. There is evidence that oocyte quality declines with increasing maternal age at conception, especially after age 30 (Van Noord-Zaadstra *et al*, 1991), owing to quality loss of its mitochondria (Papa, 1996; Ozawa, 1997). It is also known that mitochondria are passed on from mother to child. This might suggest a threshold effect rather than a linear relation with age. Nevertheless, we found no evidence for a threshold effect: for women over age 30 at first delivery, the risk of breast cancer in their daughters was lower than in younger mothers, but not statistically significantly. We hypothesised that the viability of mitochondria depends on grandmaternal age. We adjusted for many known and several less well-known risk factors for breast cancer, but the number of patients with complete information on birth dates limited the power of our study, and preventing subgroup analyses on modification by menopausal status, birth order or family history. Of the four largest cohort studies, three suggested a positive association between maternal age and breast cancer risk in daughters (Holmberg *et al*, 1995; Mogren *et al*, 1999; Xue and Michels, 2007).

Although we were able to adjust for most of the known risk factors of breast cancer, we were not able to correct the results for physical activity and oral contraceptive use because these variables had a large number of missing values.

Our study has several strengths. We used documented information about birth data instead of recalled information to calculate (grand)mothers’ age at childbirth. In addition, the main purpose of the DOM study was to evaluate the risk factors of breast cancer, thereby allowing the possibility of adjusting for most of these, whereas five of the seven other cohort studies were unable to adjust for several important adult risk factors.

In summary, in this prospective follow-up study over an 18-year period, no significant association between (grand)maternal age at delivery and breast cancer risk of their (grand)daughter was found.

## Figures and Tables

**Table 1:** Distribution of personal characteristics across levels of (grand)maternal age at delivery in the DOM study at baseline in 1982–85 (*n* participants with known maternal age=14 660 person-years, *n* participants with known grandmaternal age=7938 person-years)

	**Maternal age at delivery (years)**					**Grandmaternal age at delivery (years)**				
**Personal characteristics**	**<25**	**25–29**	**30–34**	**35–39**	**⩾40**	**<25**	**25–29**	**30–34**	**35–39**	**⩾40**
Age (year): mean	46.6	46.2	46.6	46.4	46.4	46.4	46.6	46.1	46.5	45.7
Birth weight (g): mean	3563	3467	3227	3320	3514	3111	3385	3439	3415	3246
Premature (%)	8.6	13.5	10.7	10.2	14.3	8.8	12.7	13.1	11.4	30.0
Firstborn (%)	65.6	43.6	18.0	7.4	4.2	30.6	32.5	32.0	25.5	36.0
Height (m): mean	1.65	1.66	1.66	1.65	1.65	1.65	1.66	1.66	1.64	1.65
BMI (kg m^−2^): mean	25.0	25.0	24.7	24.6	24.3	25.6	24.9	24.7	24.5	24.1
Age at first birth (years): mean	24.6	25.4	25.2	25.6	25.8	25.1	25.7	25.1	25.4	25.2
										
*Parity*										
Nulliparous (%)	12.0	12.7	12.7	18.5	6.9	8.2	16.9	7.5	16.3	10.0
Parous: mean	2.7	2.6	2.7	2.7	2.7	2.7	2.7	2.6	2.7	2.5
										
*Alcohol (%)*										
Never	19.1	15.6	19.1	18.5	16.7	22.5	22.7	16.3	12.2	14.0
Sometimes	19.1	15.0	14.6	14.8	19.4	19.4	16.2	10.9	16.3	6.0
Often	61.8	69.4	66.3	66.7	63.9	58.2	61.0	72.8	71.4	80.0
										
Dysplasia (%)	23.5	35.0	33.2	33.8	38.9	33.7	27.9	37.2	34.7	42.0
Age at menarche (years): mean	13.3	13.5	13.6	13.5	13.4	13.6	13.6	13.6	13.6	13.3
										
*Menopausal status (%)*										
Pre	13.4	21.8	18.7	11.7	5.4	13.5	21.0	19.2	12.6	7.5
Post	4.9	9.6	8.0	4.5	1.8	4.4	7.1	7.7	5.3	1.2
										
Family history positive (%)	6.0	7.0	11.6	9.3	18.1	7.2	13.3	8.3	11.6	8.3
										
Number of women included in analysis	183	314	267	162	72	98	154	147	98	50

Abbreviation: DOM=Diagnostisch Onderzoek Mammacarcinoom.

**Table 2:** Maternal and grandmaternal age at delivery *vs* the incidence of breast cancer in the (grand)daughter, during follow-up from 1982 to 2000 among participants of the DOM study

	**No. of cases**	**Person- years**	**Crude HR (95% CI)**	**Age-adjusted HR (95% CI)**	**Covariate-adjusted HR (95% CI)^a^**	**Age+covariate-adjusted^b^ HR (95% CI)**
*Maternal age (years)*			*n=998*			*n=547*
<25	53	2679	0.90 (0.61–1.33)	0.77 (0.19–3.12)	0.87 (0.59–1.28)	0.95 (0.23–3.82)
25–29.9	97	4634	1.00	1.00	1.00	1.00
30–34.9	90	3915	1.11 (0.79–1.56)	1.29 (0.25–6.57)	0.95 (0.57–1.58)	1.06 (0.42–2.68)
35–39.9	46	2389	0.87 (0.58–1.31)	1.18 (0.04–36.04)	0.72 (0.30–1.68)	0.55 (0.11–2.84)
⩾40	23	1043	1.00 (0.59–1.70)	1.58 (0.01–267.81)	0.92 (0.36–2.33)	0.47 (0.10–2.24)
*P* for trend			0.75	0.62	0.76	0.86
						
*Grandmaternal age (years)*			*n=547*			
<25	33	1423	0.89 (0.53–1.50)	0.53 (0.24–1.17)	0.86 (0.41–1.80)	0.84 (0.39–1.79)
25–29.9	55	2260	1.00	1.00	1.00	1.00
30–34.9	44	2168	0.76 (0.48–1.22)	1.24 (0.59–2.64)	0.67 (0.26–1.72)	0.71 (0.27–1.88)
35–39.9	41	1379	1.30 (0.78–2.16)	3.53 (1.00–12.41)	1.36 (0.42–4.36)	1.50 (0.46–4.86)
⩾40	24	708	1.62 (0.87–3.00)	7.29 (1.20–44.46)	1.22 (0.25–5.92)	1.45 (0.27–7.81)
*P* for trend			0.23	0.05	0.89	0.83

Abbreviations: CI=confidence interval; DOM=Diagnostisch Onderzoek Mammacarcinoom; HR=hazard ratios.

a Hazard ratio and 95% CI adjusted for alcohol, dysplasia, BMI at inclusion, no. of months breastfeeding, no. of times ⩾7 months pregnant, age at first childbirth, premature, birth weight, birth length, firstborn, HRT use, menarche, menopausal age, familial breast cancer.

b Hazard ratio and 95% CI: maternal age adjusted for grandmaternal age and covariates, grandmaternal age adjusted for maternal age and covariates.
